# The Culture of Cancer Cell Lines as Tumorspheres Does Not Systematically Result in Cancer Stem Cell Enrichment

**DOI:** 10.1371/journal.pone.0089644

**Published:** 2014-02-24

**Authors:** Christophe Y. Calvet, Franck M. André, Lluis M. Mir

**Affiliations:** 1 Univ Paris-Sud, Laboratoire de Vectorologie et Thérapeutiques Anticancéreuses, UMR 8203, Villejuif, France; 2 CNRS, Laboratoire de Vectorologie et Thérapeutiques Anticancéreuses, UMR 8203, Villejuif, France; 3 Gustave Roussy, Laboratoire de Vectorologie et Thérapeutiques Anticancéreuses, UMR 8203, Villejuif, France; Cleveland Clinic, United States of America

## Abstract

Cancer stem cells (CSC) have raised great excitement during the last decade and are promising targets for an efficient treatment of tumors without relapses and metastases. Among the various methods that enable to enrich cancer cell lines in CSC, tumorspheres culture has been predominantly used. In this report, we attempted to generate tumorspheres from several murine and human cancer cell lines: B16-F10, HT-29, MCF-7 and MDA-MB-231 cells. Tumorspheres were obtained with variable efficiencies from all cell lines except from MDA-MB-231 cells. Then, we studied several CSC characteristics in both tumorspheres and adherent cultures of the B16-F10, HT-29 and MCF-7 cells. Unexpectedly, tumorspheres-forming cells were less clonogenic and, in the case of B16-F10, less proliferative than attached cells. In addition, we did not observe any enrichment in the population expressing CSC surface markers in tumorspheres from B16-F10 (CD133, CD44 and CD24 markers) or MCF-7 (CD44 and CD24 markers) cells. On the contrary, tumorspheres culture of HT-29 cells appeared to enrich in cells expressing colon CSC markers, *i.e.* CD133 and CD44 proteins. For the B16-F10 cell line, when 1 000 cells were injected in syngenic C57BL/6 mice, tumorspheres-forming cells displayed a significantly lower tumorigenic potential than adherent cells. Finally, tumorspheres culture of B16-F10 cells induced a down-regulation of vimentin which could explain, at least partially, the lower tumorigenicity of tumorspheres-forming cells. All these results, along with the literature, indicate that tumorspheres culture of cancer cell lines can induce an enrichment in CSC but in a cell line-dependent manner. In conclusion, extensive characterization of CSC properties in tumorspheres derived from any cancer cell line or cancer tissue must be performed in order to ensure that the generated tumorspheres are actually enriched in CSC.

## Introduction

Cancer stem cells (CSC), a subpopulation of cancer cells, have raised a huge interest in the scientific community for the last two decades. They are indeed suspected to be responsible for tumor growth and metastasis, resistance toward therapy and thus relapses [1].

CSC share many features with normal stem cells such as differentiation ability, self-renewal and relative dormancy suggesting that they could possibly originate from their normal counterparts through an accumulation of transforming mutations, either genetic or epigenetic [2]–[4]. CSC are parent cells which can self-renew or differentiate into heterogeneous lineages that will form the tumor bulk. Unlike the clonal evolution model of cancer propagation, the CSC model states that all cancer cells are not equally tumorigenic when injected *in vivo* [1], [5]. Fifteen years ago, Bonnet and Dick showed for the first time that CD34^+^CD38^−^ CSC isolated from acute myeloid leukemia were able to recapitulate the heterogeneity of the original tumor through serial transplantations in xenograft models, contrary to CD34^−^CD38^+^ cells [6]. Since then, the CSC model was used to explain not only the propagation of leukemia but also of solid cancers such as breast, gastric, colon, prostate, ovarian, liver, pancreas, lung, brain and thyroid carcinomas, melanoma, osteosarcoma and Ewing sarcoma [7].

CSC display a resistance toward chemotherapy and radiotherapy. Their ability to escape the cytotoxicity of conventional anticancer therapies and to regenerate the tumor at the end of the treatments is due to several mechanisms: dormancy, expression of anti-apoptotic proteins, drug efflux pumps, high metabolism capacity and a low level of reactive oxygen species [1], [8], [9].

Moreover, as for normal stem cells, the CSC niche has been shown to play an active role in the maintenance of stemness properties as well as in the dedifferentiation of non-CSC through an epithelial-to-mesenchymal transition (EMT) [10]. This phenomenon, known to be involved in metastatic process, may also explain the origin of CSC as these cells share most of their properties with post-EMT cells, including high invasion and migration ability, and increased expression of mesenchymal markers (*e.g.* vimentin). Moreover, cancer cells forced to undergo EMT possess an increased tumorigenic potential and express higher levels of CSC markers [8], [10]–[12].

Few methods are currently available to isolate CSC. Cultivating cells in an anchorage-independent manner, into a serum-free medium enriched in growth factors, was first used to propagate human mammary epithelial cells in an undifferentiated state [13]. Ponti and co-workers showed that this method was also efficient in maintaining breast CSC in culture [14]. In such conditions, cells grew as multicellular three-dimensional clones called “tumorspheres”. This technique proved its efficiency in enriching and maintaining CSC from several cell lines [15].

In this paper, we sought to evaluate the ability of the tumorspheres culture technique to enrich several cancer cell lines in CSC. Indeed, it is of great practical interest for drug discovery and for the evaluation of the efficiency of treatments to work with cancer cell lines enriched in CSC since these are considered as the one that should be targeted to treat tumors in the long term without recurrence.

## Materials and Methods

### Ethics Statement

All animal experiments were performed in strict compliance with the ethical guidelines issued by the European Committee (Directive 86/609/CCE). The Université Paris-Sud Animal Ethics Committee #26, registered by the French Department of Research, specifically approved this protocol (protocol registration number #2012_007).

### Adherent Cell Culture

B16-F10 murine melanoma, HT-29 human colon adenocarcinoma, MCF-7 and MDA-MB-231 human breast adenocarcinoma cell lines were cultivated in DMEM, McCoy’s 5A, MEM or RPMI 1640 medium, respectively. All media were supplemented with 1% glutamax, 10% fetal bovine serum (FBS), 100 U/mL penicillin and 100 µg/mL streptomycin (PS), all purchased from Life technologies (Cergy Pontoise, France). Insulin solution (Sigma, St Quentin Fallavier, France) at a 10 µg/mL final concentration was specifically used for MCF-7 cell culture. Cells were propagated at 37°C in a 95% humidity atmosphere containing 5% CO_2_ and passaged upon confluency (at a 1∶10, 1∶8, 1∶6 or 1∶4 dilution, respectively) using a TrypLE solution (Life technologies). Cells were mycoplasma-free and routinely checked with the Venor GeM-One Step Mycoplasma detection kit purchased from Biovalley (Marne-la-Vallée, France).

### Tumorspheres Culture

10 000 viable cells were transferred in a T75 ultra-low adherence flask (Corning, Avon, France) in 10 mL of CSC medium consisting in DMEM/F12 medium plus glutamax (Life technologies), 4 µg/mL heparin (Sigma), 2% B27 supplement (Life technologies), 20 ng/mL epidermal growth factor (EGF, Peprotech, Neuilly-sur-Seine, France), 20 ng/mL basic fibroblast growth factor (FGF-b, Peprotech) and 1% PS. Fresh EGF, FGF-b and heparin were added to the medium every 3 days. Tumorspheres were allowed to grow during 4 days (B16-F10) or 7 days (HT-29 and MCF-7). For dissociation, tumorspheres were first centrifuged for 5 minutes at 200 g, the pellet was then gently resuspended in 500 µL of Accutase (Life technologies) before being incubated for 5 minutes at 37°C. After adding 2 mL of DMEM/F12, tumorspheres were dissociated by gentle pipetting and directly transferred back into tumorspheres culture conditions as previously described.

### Tumorsphere-forming Efficiency Assay

ARIAIII Cell sorter (BD biosciences, USA) was used to precisely transfer 1 single viable cell (*i.e.* propidium iodide-negative cell) into each well of an ultra-low adherence 96-well plate (Corning) containing 100 µL of CSC medium. Fresh EGF, FGF-b and heparin were added every 3 days. This experiment was performed in order to assess the tumorsphere formation ability of cells originating from adherent cultures or from previously formed primary or secondary tumorspheres. After 10 days of culture, the number of wells containing a tumorsphere larger than 50 µm was determined using a phase contrast microscope.

### Clonogenic Assay

ARIAIII Cell sorter was used to precisely transfer 200 viable cells (*i.e.* propidium iodide-negative cells), dissociated from one-week-old tumorspheres or from adherent monolayers culture, into each well of a normal adherence 6-well plate containing DMEM supplemented with 10% FBS and 1% PS. After 5 days of culture for B16-F10 and 10 days for HT-29 and MCF-7, medium was discarded, cells were washed with phosphate buffered saline (PBS) and fixed and stained using an aqueous solution containing 20% ethanol, 3.7% formaldehyde and 0.2% crystal violet. Clonogenicity was calculated as the number of colonies formed relative to the number of colonies formed from adherent cells.

### Proliferation Assay

ARIAIII Cell sorter was used to transfer 1 000 B16-F10 viable cells (*i.e.* propidium iodide-negative cells), dissociated from one-week-old tumorspheres or from adherent monolayers, into each well of a normal adherence 96-well plate containing DMEM supplemented with 10% FBS and 1% PS. The plate was placed into an IncuCyte™ FLR imaging system (Essen Biosciences, Welwyn Garden City, UK) within a regular cell culture incubator (37°C, 95% humidity, 5% CO_2_). Four different areas per well were monitored (10× magnification, phase contrast) with the IncuCyte™ every 4 hours during one week. Proliferation was measured with the IncuCyte™ software and the doubling time was determined using a linear regression model.

### Immunostaining Assay

50 000 viable B16-F10 cells (trypan blue exclusion test) dissociated from one-week-old tumorspheres or from trypsinized adherent monolayers were incubated for 30 minutes at 4°C in the dark with 0.5 µg of rat anti-mouse monoclonal CD133-APC, CD44-FITC or CD24-PE antibodies (eBiosciences, Paris, France) in 100 µL of a buffer solution consisting in PBS containing 3% bovine serum albumin (Sigma). Immunostaining was also performed for HT-29, MCF-7 and MDA-MB-231 cell lines using mouse anti-human monoclonal CD133-APC, CD44-PE, CD44-APC or CD24-FITC antibodies (Miltenyi Biotec, Paris, France) according to the manufacturer’s instructions. Cells were then washed and analyzed with an Accuri C6 flow cytometer (BD biosciences, USA).

### Side Population Assay

A single cell suspension of 1.10^6^ B16-F10 cells/mL in a serum-free DMEM/F12 medium was prepared using dissociated one-week-old tumorspheres or adherent monolayers. This cell suspension was incubated with 5 µg/mL Hoechst 33342 (Sigma) for 90 minutes at 37°C in the dark. A negative control cell suspension exposed to Hoescht 33342 and 50 µM verapamil (Sigma) was incubated in parallel. Cells were then washed with PBS and resuspended in a serum-free DMEM/F12 solution containing 5 µg/mL propidium iodide (Sigma) to exclude dead cells. Analysis was performed using a LSR II flow cytometer (BD biosciences).

### Quantitative Reverse Transcription Polymerase Chain Reaction (RT-qPCR)

TRIzol® reagent (Life technologies) was used to extract total RNA from B16-F10 adherent cells or tumorspheres according to the manufacturer’s instructions. 1 µg of RNA was reverse-transcribed using M-MLV reverse transcriptase (Life technologies). Amplification and detection by SYBR Green was realized using the Step One Plus Real Time PCR System (Applied Biosystems, France) according to the manufacturer’s guidelines. 18S housekeeping gene was used as an internal standard. The following primers were used at 10 µM each:

E-cadherin:

forward 5′-GAGCCTGAGTCCTGCAGTCC-3′, reverse: 5′-TGTATTGCTGCTTGGCCTCA-3′

Vimentin:

forward 5′-CACCCTGCAGTCATTCAGACA-3′, reverse: 5′-GATTCCACTTTCCGTTCAAGGT-3′

Snai1:

forward 5′-GGAAGCCCAACTATAGCGAGC-3′, reverse: 5′-CAGTTGAAGATCTTCCGCGAC-3′

18S:

forward 5′-GTAACCCGTTGAACCCCATT-3′, reverse: 5′-CCATCCAATCGGTAGTAGCG-3′

### Tumorigenicity Assay

100 µL of DMEM/F12 containing 300, 1 000, 3 000, 10 000, 30 000 or 100 000 viable cells (trypan blue exclusion test) from dissociated B16-F10 tumorspheres were injected in both flanks of 6-to-8-week-old C57BL/6 mice (Harlan, Gannat, France) at two different locations. The 1 000 cells-injected mice group comprised 6 mice and the other groups comprised 3 mice each. The control groups were injected with the same numbers of cells originating from B16-F10 adherent cultures and resuspended in 100 µL of DMEM. Mice were checked two to three times a week during 50 days. Mice were sacrificed as soon as the tumors reached 2 000 mm^3^ or became necrotic in order to minimize animal suffering.

### Statistical Analysis

Data are presented as means and standard deviations.

Data were analyzed using non parametric Mann-Whitney-Wilcoxon test, Kruskall-Wallis test with Dunn’s correction or χ2 test and p<0.05 was considered statistically significant.

## Results

### B16-F10, HT-29 and MCF-7 Cells are able to form Tumorspheres

**Figure 1:**
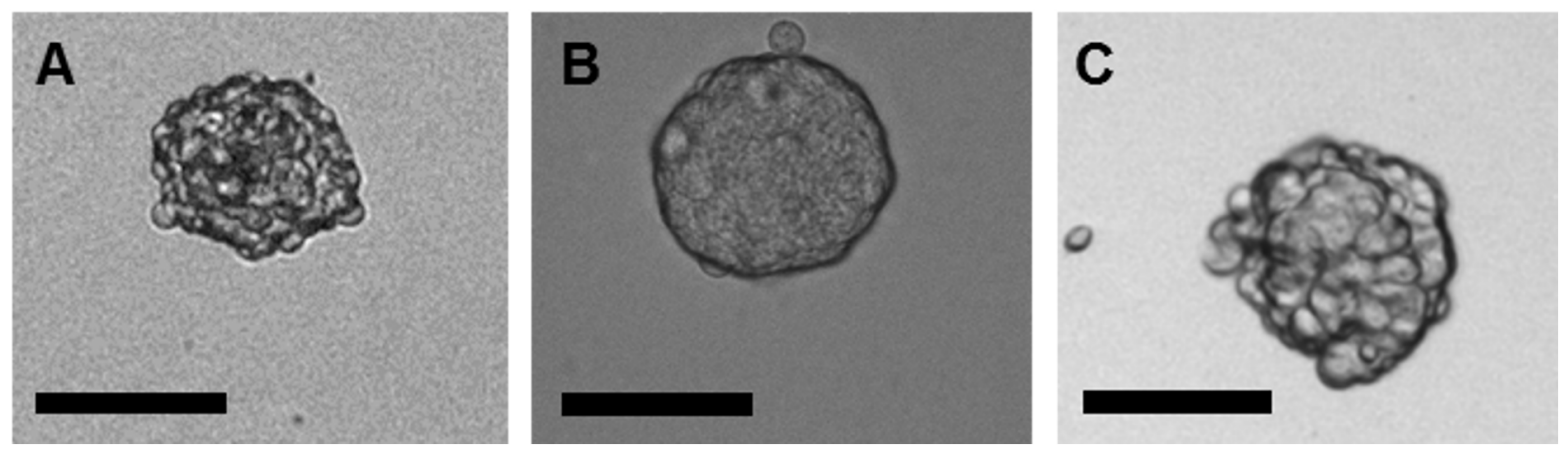
Microscopy pictures presenting the morphology of tumorspheres. (A) B16-F10, (B) HT-29 and (C) MCF-7 tumorspheres. Tumorspheres were formed after 4 to 7 days of culture in serum-free medium containing FGF-b and EGF on ultra-low adherence substrate. Scale bars represent 100 µm.

B16-F10, HT-29, MCF-7 and MDA-MB-231 cell lines were cultivated in an anchorage-independent manner, which means onto a non-adherent substrate in a serum-free medium containing growth factors aiming to sustain pluripotency (*i.e.* FGF-b and EGF). After 4 days in culture for B16-F10 cells and 7 days for HT-29 and MCF-7 cells, tumorspheres of approximately 100 µm in diameter were observed (Figure 1) and dissociated for subculture. On the contrary, MDA-MB-231 cells did not manage to form tumorspheres, even after more than 10 days.

**Figure 2:**
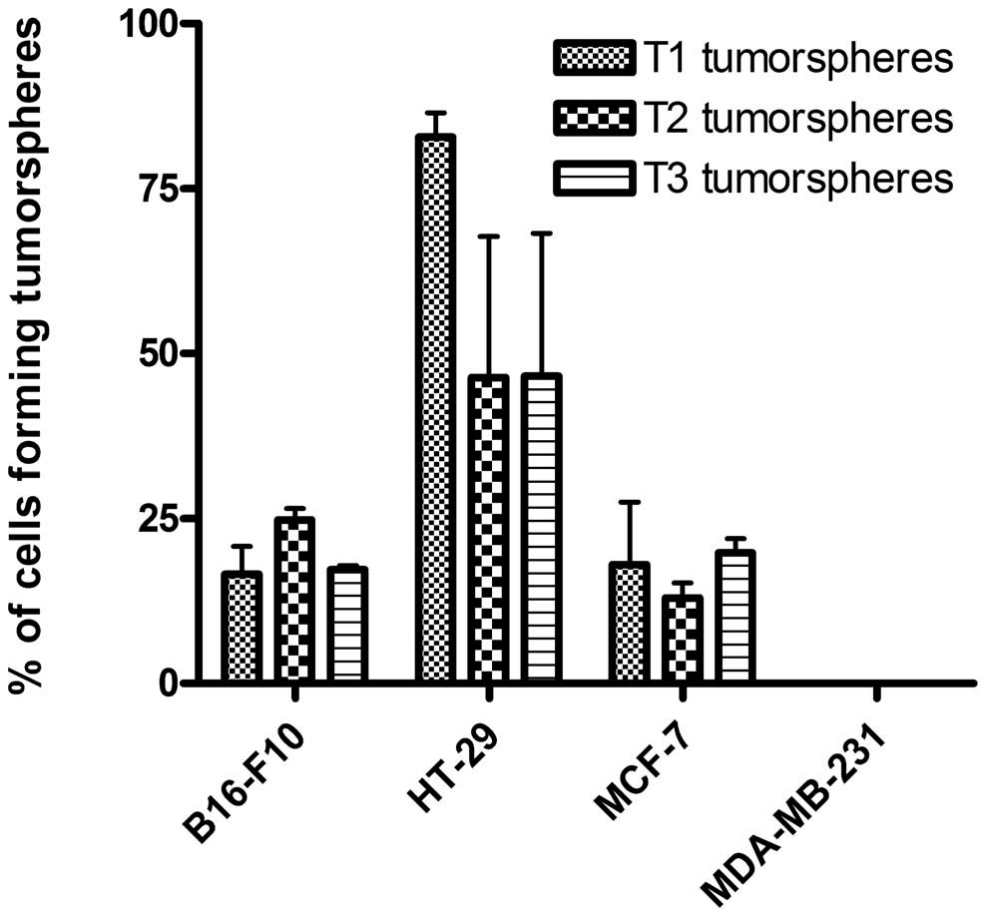
Tumorspheres formation rates over the three first passages as tumorspheres. Tumorspheres formation efficiencies were highly cell line-dependent. T1: primary, T2: secondary, T3: tertiary.

B16-F10 tumorspheres were able to be cultured as tumorspheres for extended passages (more than 20 passages). About 20% of the B16-F10 cells were able to form a tumorsphere and this rate remained relatively constant over at least the three first passages (Figure 2). Similar rates were obtained for MCF-7 tumorspheres formation efficiency. Regarding the HT-29 cell line, 80% of the adherent cells were able to form primary tumorspheres while we observed a decrease down to about 50% of the cells for secondary or tertiary tumorspheres formation. However, these differences were not statistically significant.

### The Ability to form Adherent Colonies is Decreased after One Week of Culture as Tumorspheres

**Figure 3:**
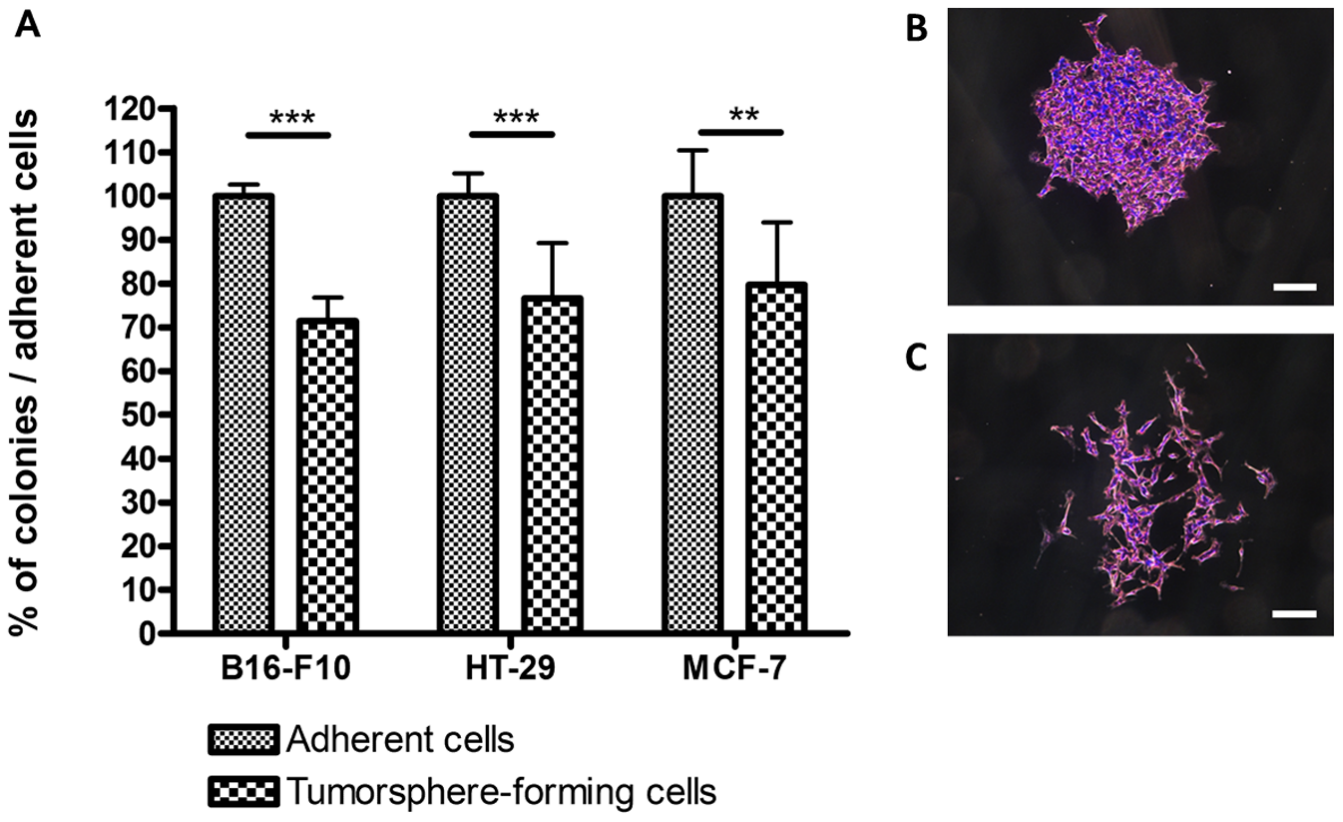
Comparison of the ability to form adherent colonies of the tumorspheres-forming and the adherent cells. (A) Tumorspheres-forming cells generated approximately 20 to 30% less colonies than adherent cells, depending on the cell line considered (*** for p<0.001 for B16-F10 and HT-29 cells, ** for p<0.01 for MCF-7 cells). (B) Adherent B16-F10 cells always formed round-shaped and packed colonies whereas (C) B16-F10 tumorspheres-forming cells also formed a variable number of poorly dense colonies, ranging from 30% up to 80% of the total number of colonies. Scale bars represent 200 µm.

One-week-old tumorspheres derived from B16-F10, HT-29 or MCF-7 cells were dissociated, transferred back to adherent conditions and their colony formation efficiencies compared with that of adherent cells. Clonogenicity of tumorspheres-forming cells derived from the three cell lines was significantly reduced, namely a 20-to-30% drop compared to adherent cells (Figure 3, panel A). Moreover, 30% to 80% of adherent colonies derived from B16-F10 tumorspheres-forming cells were visibly less dense than the ones resulting from adherent cells (Figure 3, panels B and C). This suggests that tumorspheres-forming cells underwent an adaptation or a selection to the culture in suspension, resulting in a weaker capacity to attach to the substrate. No such change in colonies morphology was observed in the case of the HT-29 and MCF-7 cell lines.

### B16-F10 Tumorspheres-forming Cells Grow more Slowly in Adherent Conditions than their Adherent Counterparts

**Figure 4:**
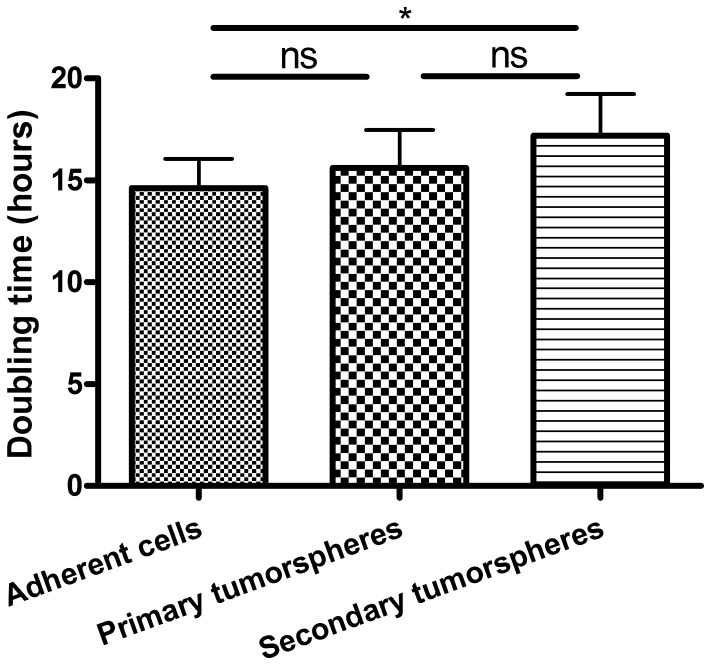
Comparison of doubling times of B16-F10 adherent and tumorspheres-forming cells both cultured under adherent conditions. Doubling time of B16-F10 cells from secondary tumorspheres was significantly higher than that of adherent cells. Ns: not statistically significant, * for p<0.05.

B16-F10 adherent and tumorspheres-forming cells (both from primary and secondary tumorspheres) were cultured under adherent conditions for one week and their proliferation was quantified using the Incucyte™ confluence algorithm. Doubling time of B16-F10 cells from secondary tumorspheres was significantly higher than that of their adherent cell counterparts (Figure 4) suggesting again that tumorspheres-forming cells underwent an adaptation to the culture in suspension, resulting in a weaker proliferation under adherent conditions.

### Tumorspheres Culture Affects the Expression of CSC Markers in a Cell Line-dependent Manner

Immunostaining is also a classical method to identify CSC [1], [15]. Therefore, cells from tumorspheres or adherent monolayers were immunostained using antibodies recognizing the most common CSC surface markers, *i.e.* CD133, CD44 and CD24 proteins (Table 1). According to the literature, CD133, CD44 and CD24 are used to identify CSC population in B16-F10 cell line [16] whereas only CD133 and CD44 are identified as reliable markers for HT-29 CSC [17]. Regarding breast cancer cell lines such as MCF-7 and MDA-MB-231, a CD44^+^CD24^−^ profile was described for CSC [18], [19].

**Table 1:** Percentages of cells expressing the CSC markers in adherent and tumorspheres-forming cells.

CSC population	Adherent cells	Tumorspheres-forming cells
**CD133^+^CD44^+^CD24^+^ B16-F10**	<1%	<1%
**CD133^+^CD44^+^ HT-29**	34.3% +/−11.3%	49.6% +/−4.8%
**CD44^+^CD24** ^−^ **MCF-7**	50.6% +/−3.3%	33.1% +/−5.1%
**CD44^+^CD24** ^−^ **MDA-MB-231**	99.7% +/−0.4%	NA

NA: not applicable.

In adherent monolayers, almost all B16-F10 cells expressed CD44 whereas CD133 and CD24 proteins were found in less than 1% of the cells. However, these rates remained unchanged after cultivating the cells as tumorspheres for up to 23 passages. Although CSC can also be identified as a side population able to efflux Hoechst 33342 thanks to membrane ABC transporters [15], [20], no side population was detected in either B16-F10 adherent or tumorspheres-forming cells (data not shown).

Regarding the HT-29 cell line, a 15% increase of CD133^+^CD44^+^ cells was detected in tumorspheres (49.6% of cells) compared to adherent cells (34.3% of cells), hinting towards an enrichment in CSC. However, this difference was not statistically significant because of high standard deviations.

The MCF-7 cells contained about 50% CD44^+^CD24^−^ cells in the adherent population. This rate decreased down to 33.1% when cells were cultivated as tumorspheres, although this difference was not statistically significant. In the case of the MDA-MB-231 cells, which were not efficient at all in forming tumorspheres, more than 99% of the adherent cell population was CD44^+^CD24^−^.

### Tumorspheres Culture of B16-F10 Decreases the Expression of Vimentin and E-cadherin

**Figure 5:**
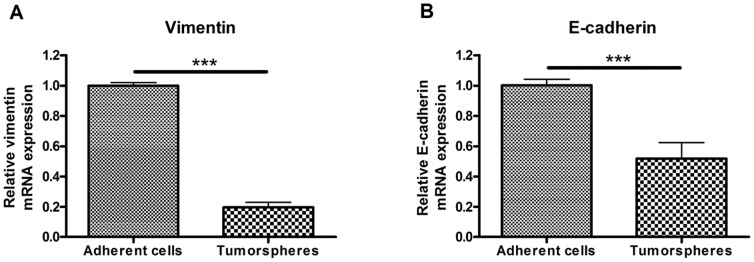
Relative expression of vimentin and E-cadherin in both B16-F10 adherent and tumorspheres-forming cells. Both vimentin (A) and E-cadherin (B) were down-regulated in tumorspheres. *** for p<0.001.

As mentioned earlier, several authors reported that CSC possess post-EMT cells characteristics, including the down-regulation of epithelial markers (*e.g.* E-cadherin) and the up-regulation of mesenchymal markers (*e.g.* vimentin and Snai1) [8], [11]. A RT-qPCR analysis was performed in order to compare the expression of E-cadherin, vimentin and Snai1 in both B16-F10 adherent cells and tumorspheres. Both E-cadherin and vimentin gene expression were significantly decreased when cells were cultured as tumorspheres. We detected 50% less E-cadherin mRNA (Figure 5, panel A) and 80% less vimentin mRNA in tumorspheres than in adherent cells (Figure 5, panel B). Snai1 mRNA was not detected in either cell culture conditions.

### B16-F10 Tumorspheres-forming Cells are less Tumorigenic than Adherent Cells in a Syngenic Mouse Model

**Figure 6:**
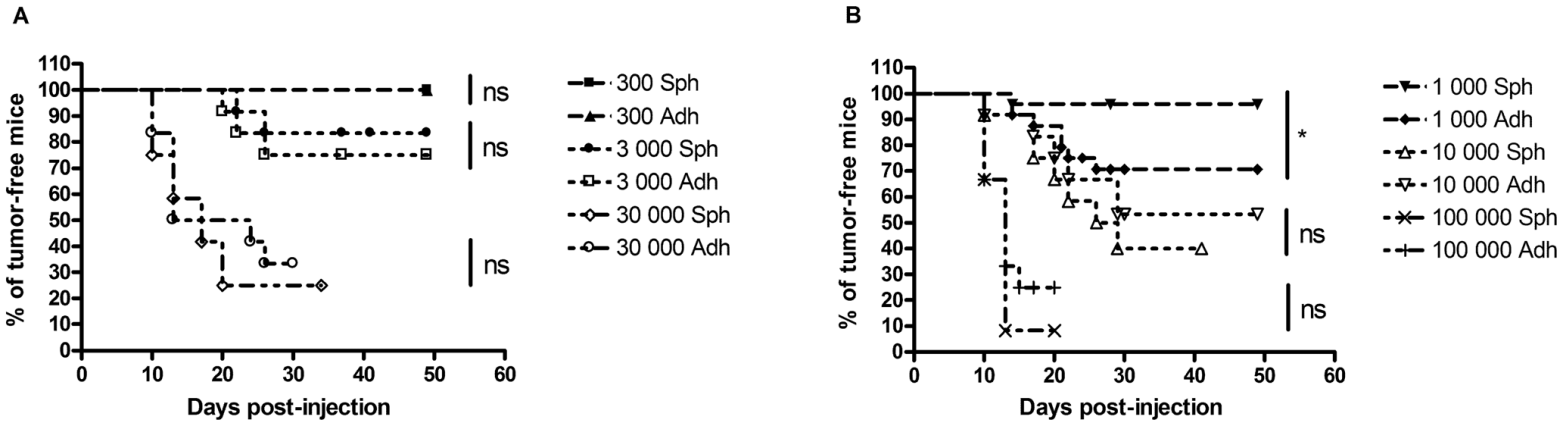
Comparison of tumor occurrence after injection of variable number of B16-F10 adherent or tumorspheres-forming cells. (A) Injection of 300, 3 000 or 30 000 cells. (B) Injection of 1 000, 10 000 or 100 000 cells. When 1 000 cells were injected in mice, tumorspheres-forming cells were significantly less tumorigenic than their adherent counterparts. Adh: adherent cells, Sph: tumorspheres-forming cells. Ns: not significant, * for p<0.05.

The gold standard method to evaluate the presence of CSC consists in injecting a CSC enriched population in mice and observing higher tumor take rates compared to mice injected with non-CSC enriched cells [1], [15]. C57BL/6 mice were injected sub-cutaneously with either 300, 1 000, 3 000, 10 000, 30 000 or 100 000 B16-F10 cells which were adherent cells for one group and tumorspheres-forming cells for the second group. Tumors were detected when at least 1 000 cells had been injected. When this number of cells was injected, tumorspheres-forming cells were significantly less tumorigenic than their adherent counterparts (Figure 6, panel B). However, at any of the higher amounts of injected cells, no significant difference was observed between the two groups (Figure 6, panel A and B).

## Discussion

Cancer stem cells (CSC) are currently extensively studied since they are supposed to initiate and sustain tumor growth and moreover are thought to be responsible for tumor recurrence [1].

Tumorspheres culture has been reported to enrich several cancer cell lines such as breast, liver, colon and ovarian cancer cell lines in CSC [15]. In this paper, we tested the CSC enrichment by tumorspheres culture of B16-F10 murine melanoma cells, HT-29 human colon adenocarcinoma cells, and MCF-7 and MDA-MB-231 human breast adenocarcinoma cells.

As opposed to what Zhong *et al.* observed [21], our experiment showed that B16-F10 cells proliferated easily in suspension as tumorspheres during 3 months at least. The tumorspheres-forming rates of B16-F10 and MCF-7 cells remained constant over the three first passages, which is an accepted hallmark of CSC self-renewal. HT-29 cells also generated tumorspheres although the formation rate tended to decrease after the first passage. On the contrary, the tumorspheres culture of MDA-MB-231 cells remained unsuccessful. This observation was consistent with what some authors reported [22], [23], although others showed this cell line was actually able to form tumorspheres [24], [25]. We hypothesized that the extinction of the tumorspheres formation ability of our MDA-MB-231 cells could result from the fact that these cells had been passaged many times before our experiments. Indeed, this consequence of the cell passage on tumorspheres formation rates has been previously demonstrated [26]. Further characterizations were therefore performed so as to conclude on the stemness properties of B16-F10, HT-29 and MCF-7 tumorspheres.

The CSC concept states that the tumor growth is driven by cancer cells with stemness characteristics which have acquired a proliferative potential and a clonogenicity mediated by a self-renewal ability. In agreement with this hypothesis, several reports mention that putative CSC possess enhanced clonogenicity [27]–[31] and proliferate more rapidly [29]–[31] than non-CSC. However, our study showed that B16-F10 tumorspheres-forming cells were less proliferative than adherent cells in adherent conditions. In addition, B16-F10, HT-29 and MCF-7 cells displayed a lower clonogenic potential when cultured as tumorspheres. We hypothesized that tumorspheres-forming cells adapted to the culture in suspension, resulting in a weaker ability to attach to (and grow on) a regular adherent substrate. This somehow rules out the description of an enrichment in CSC in the tumorspheres.

We further investigated stemness characteristics using classical biomarkers reported for CSC identification [1], [15]. Dou and co-workers showed that B16-F10 CD133^+^CD44^+^CD24^+^ cells are enriched in CSC [16]. Therefore, we compared the percentage of CD133^+^, CD44^+^ and CD24^+^ cells in B16-F10 tumorspheres and adherent cells but we did not observe any difference. Accordingly, even though Hoechst 33342 efflux-competent cells have also been shown in the literature to be enriched in putative CSC [15], [20], no such cell population was detected in both tumorspheres and adherent B16-F10 cells. This suggests that the side population assay is not relevant for the detection of CSC in all cell types.

Regarding the HT-29 cell line, one third of the population had the profile of colon CSC, *i.e*. were CD133^+^CD44^+^ cells [17], [32], and this rate rose up to 50% when cells were cultured as tumorspheres. However, due to high standard deviations in this assay along with the observations of a decreased clonogenicity and a decreased tumorspheres formation efficiency after the first passage, we could not conclude on a CSC enrichment.

We also quantified the CD44^+^CD24^−^ breast CSC population [33] in both MCF-7 and MDA-MB-231 cells. The former cell line displayed a 17% decrease in CD44^+^CD24^−^ cell population in tumorspheres, consistently with the lower clonogenic potential we observed when cells were cultured as such. Surprisingly, almost all MDA-MB-231 adherent cells were CD44^+^CD24^−^, although we did not obtain any tumorsphere from this cell line.

All these data suggest that the formation of tumorspheres does not always correlate with an enrichment in previously described CSC markers and also that the proportion of cells expressing these CSC markers does not predict the tumorspheres formation ability, at least in the frame of the cancer cell lines studied in this report.

More strikingly, the tumorigenic potential of the B16-F10 tumorspheres-forming cells was lower than that of the B16-F10 adherent cells, confirming all the *in vitro* data we obtained. Very recently, Collura and co-workers published similar findings [34]. However, since tumorspheres were also found to be more efficient in initiating tumors than adherent monolayers in the case of several other cancer cell lines [35]–[38], this suggests that the effect of tumorspheres culture on tumorigenicity strongly depends on the studied cell line.

An increasing number of authors report that CSC present post-EMT cells characteristics [8], [10]–[12]. The expression of three EMT-related genes (coding for E-cadherin, Snai1 and vimentin) was evaluated in B16-F10 tumorspheres using RT-qPCR analysis. E-cadherin is involved in interactions between epithelial-like cells and is down-regulated by Snai1. Vimentin is an intermediate filament expressed in mesenchymal cells. The two major hallmarks of EMT are a down-regulation of E-cadherin expression and an up-regulation of vimentin expression [39]–[42]. In tumorigenicity assays, this leads to an increased tumor take. In our study on B16-F10 cells, vimentin mRNA level was dramatically decreased in tumorspheres, supporting the fact that they presented a lower tumorigenicity compared to adherent cells. However, E-cadherin mRNA level was also lower in tumorspheres and, surprisingly, we did not detect Snai1 mRNA which triggers the down-regulation of E-cadherin during EMT [8]. This shows that another pathway may be involved in tumorspheres-forming cells in order to prevent the expression of E-cadherin. The combination of the decreased expression of E-cadherin, thus promoting EMT, along with the decreased expression of vimentin, promoting a mesenchymal-to-epithelial transition (MET), may explain the small difference of tumorigenicity between tumorspheres and adherent cells. Our findings show that tumorspheres culture of B16-F10 cells indeed affects the expression of EMT-related genes but it remains to be elucidated if the cells in tumorspheres present the characteristics of EMT or MET.

First, we conclude that strong expression of CSC surface markers is not predictive of tumorspheres formation, as shown for MDA-MB-231 cells.

Second, our results lead us to conclude that tumorspheres culture is not an efficient method to enrich B16-F10 murine melanoma and MCF-7 human breast adenocarcinoma cell lines in CSC. Regarding the HT-29 cell line, the results were less clear and no conclusion could be drawn. Considering our study and the others reporting an enhancement of CSC traits in tumorspheres cultures, we conclude that this method appears to enrich in CSC in a cell line-dependent manner, regardless of the species or the cancer types considered. Although our conclusions are drawn only from cancer cell lines, tumorspheres generated from primary human tumors (gliomas) have also been reported to be difficult to subculture with increased differentiation and apoptosis compared to adherent CSC culture on laminin coated flasks [43].

To summarize, the formation of tumorspheres does not always predict an enrichment in CSC and thus cannot be considered as a universal method for CSC enrichment in cancer cell lines. An extensive characterization for CSC signature in tumorspheres-forming cells is foremost mandatory before concluding on the achievement of the CSC enrichment.

Outside the frame of CSC enrichment, the generation of tumorspheres remains an interesting and useful technique. For example, it has been demonstrated several years ago that DNA transfection efficiency in tumor is low [44], [45] due to a very complex structure and that only cells located on the outer surface of the tumor are easily accessible and thus efficiently transfected [46]. Tumorspheres might be considered as an easy, rapid and non-animal-depending model to test further improvements of gene delivery in tumors.
